# Olive Mill and Winery Wastes as Viable Sources of Bioactive Compounds: A Study on Polyphenols Recovery

**DOI:** 10.3390/antiox9111074

**Published:** 2020-11-01

**Authors:** Paulina Tapia-Quirós, Maria Fernanda Montenegro-Landívar, Monica Reig, Xanel Vecino, Teresa Alvarino, Jose Luis Cortina, Javier Saurina, Merce Granados

**Affiliations:** 1 Chemical Engineering Department, Escola d’Enginyeria de Barcelona Est, Universitat Politècnica de Catalunya (UPC)-Barcelona TECH, 08930 Barcelona, Spain; paulina.tapia@upc.edu (P.T.-Q.); maria.fernanda.montenegro@upc.edu (M.F.M.-L.); monica.reig@upc.edu (M.R.); xanel.vecino@upc.edu (X.V.); jose.luis.cortina@upc.edu (J.L.C.); 2 Barcelona Research Center for Multiscale Science and Engineering, 08930 Barcelona, Spain; 3 Galician Water Research Center Foundation (Cetaqua Galicia), University of Santiago de Compostela, E-15782 Santiago de Compostela, Spain; teresa.alvarino@cetaqua.com; 4 CETAQUA, Carretera d’Esplugues, 75, 08940 Barcelona, Spain; 5 Department of Chemical Engineering and Analytical Chemistry, Universitat de Barcelona, Diagonal 645, 08028 Barcelona, Spain; xavi.saurina@ub.edu

**Keywords:** olive pomace, wine lees, phenolic compounds, circular economy, ethanol–water, ultrasound assisted extraction, microwave assisted extraction, pressurized liquid extraction

## Abstract

In this study, the recovery of polyphenols from olive oil mill and winery waste was investigated. The performance of ultrasound assisted extraction (UAE), microwave assisted extraction (MAE), and pressurized liquid extraction (PLE) was assessed using ethanol–water mixtures, which are compatible with food, nutraceutical, and cosmetic applications. The extraction efficiency from olive pomace and lees samples was evaluated in terms of total polyphenol content (TPC), determined by high performance liquid chromatography (HPLC) and Folin–Ciocalteu assay. The effect of solvent composition, temperature, and time was analyzed by response surface methodology. Ethanol:water 50:50 (*v/v*) was found to be a suitable solvent mixture for both kinds of samples and all three extraction techniques. The performance of the extraction techniques was evaluated, under optimal experimental conditions, with a set of different representative samples of residues from olive oil and wine production. Overall, the best extraction efficiency for olive pomace residues was provided by MAE (ethanol:water 50:50 (*v/v*), 90 °C, 5 min), and for wine residues by PLE (ethanol:water 50:50 (*v*/*v*), 100 °C, 5 min, 1 cycle). However, the results provided by UAE (ethanol:water 50:50 (*v*/*v*), 30 min) were also suitable. Considering not only extraction performance, but also investment and operational costs, UAE is proposed for a future scaling up evaluation. Regarding olive pomace as a source for natural phenolic antioxidants, olive variety and climatic conditions should be taken into account, since both influence TPC in the extracts, while for winery residues, lees from red wines are more suitable than those from white wines.

## 1. Introduction

Olive oil and wine industries generate large amounts of solid and liquid wastes. In the production of olive oil, one ton of olives generates about 0.40 ton of olive pomace, which is composed of fragments of olive skin, pulp, and bone [1,2], and 1200 L of olive mill waste water, depending on the oil extraction process [3]. On the other hand, as a result of the wine production, one ton of grapes generates approximately 0.06 ton of lees, among other residues. Lees mainly consist of yeasts and bacteria [4,5]. 

These wastes are harmful to the environment but exceptionally rich in bioactive compounds, such as polyphenols [3]. Polyphenols are secondary metabolites of plants, characterized by the presence of more than one phenol group per molecule. They mainly prevent the formation of free radicals involved in oxidation processes, donating hydrogen atoms or electrons [6]. The strong antioxidant potential of these molecules is of particular interest to the food, cosmetic, and pharmaceutical industries because of their benefits for human health [7]. As olive oil and wine production are major economic activities in Southern Europe, it is worthwhile to delve deeply into the recovery of these active compounds from production wastes and by-products. For instance, about 5000 kton/year of olive pomace and 3500 kton/year of wine residues are produced in Spain, with an average TPC of 900 g/ton and 450 g/ton respectively [8,9]. Thus, the recovery of polyphenols constitutes a key point for the valorization of these materials and would support a more sustainable bioeconomy, reducing environmental problems caused by these wastes and making them suitable for commercialization [7].

Extraction is the first step for the recovery of polyphenols. Various techniques can be applied, ranging from simple mechanical agitation to others that use some type of additional energy, such as ultrasound assisted extraction (UAE), microwave assisted extraction (MAE), or pressurized liquid extraction (PLE) [10]. UAE consists of the application of high frequency waves (≥2 MHz) that allow the formation of cavitation bubbles and the rupture of the cell membranes in the sample, improving the transport between the solid matrix and the liquid phase. UAE can be done in an ultrasonic bath or with an ultrasonic probe [7,11]. MAE uses microwave energy, which can be absorbed by polar molecules. Thus, MAE mostly uses polar solvents, whereas the bulk system is heated; moreover, the increase of the pressure inside the vegetal cells contributes to the rupture of the cell wall, which facilitates the extraction process. MAE is mostly performed with closed vessels [7,10,11,12]. PLE is based on the use of solvents at high pressure [13,14]. This allows to perform the extraction process at temperatures higher than the standard boiling temperature of the solvent, while the solvent is in liquid state. High pressure combined with high temperature improves both the kinetics of the mass transfer, and the efficiency of the extraction system [11,15]. 

UAE, MAE, and PLE have been shown to be suitable techniques for the extraction of polyphenols from samples of vegetable origin, including different olive oil [14,16,17,18,19,20,21,22,23,24,25,26,27], and winemaking [28,29,30,31,32,33,34,35,36,37,38,39,40,41] residues. Compared to conventional extraction techniques, higher extraction efficiency, shorter extraction time, or lower solvent consumption have been highlighted [10]. 

Regardless of the extraction technique, solvent composition is a key factor for successful extraction. For the extraction of polyphenols from olive pomace samples, isopropanol [22], methanol [16], ethanol [19,25], and water [24] have been used, whereas for wine lees samples, ethanol and water [28,31] have been mostly proposed. When the final application of the extracts is for their use as food additives, solvents such as water or ethanol are the best option, because of their compatibility with the food industry [14,35].

This study is part of a long-term research project aimed at the development of polyphenol recovery and purification processes. In particular, this work is focused on (i) identifying suitable wastes from the olive oil and wine production sectors, and (ii) selecting the more efficient extraction stage, considering recovery yields as well as capital and operation expenditures. Thus, the aim of this work is to explore the performance of the water–ethanol system in the extraction of polyphenols by UAE, MAE, and PLE, and to characterize TPC and antioxidant activity in several wastes from olive oil and wine production, considering different varieties of olive or grape.

## 2. Materials and Methods 

### 2.1. Reagents

Polyphenols standards: rutin, gallic acid, 3,4-dihydroxybenzoic acid, chlorogenic acid, vanillic acid, syringic acid, ethyl gallate, ferulic acid, 3,4-dihydroxybenzaldeyde, 4-hydroxybenzoic acid, epicatechin, *p*-coumaric acid, naringenin, quercetin, 2,5-dihydroxybenzoic acid, and apigenin were obtained from Sigma-Aldrich (St. Louis, MO, USA); 3-hydroxytyrosol, catechin, resveratrol, and myricetin from TCI (Tokyo, Japan); homogentisic acid and oleuropein from Extrasynthese (Lyon, France); kaempferol and hesperidin from Glentham Life Sciences (Corsham, UK); caffeic and caftaric acid from Chengdu Biopurify Phytochemicals (Chengdu, China); luteolin and 6-hydroxy-2,5,7,8-tetramethylchroman-2-carboxylic acid (trolox) from Carbosynth (Berkshire, UK). 

Reagents for antioxidant indexes were Folin–Ciocalteu reagent from Panreac (Barcelona, Spain), potassium peroxydisulfate from Merck (Darmstadt, Germany), and 2,2′-azino-bis(3-ethylbenzothiazoline-6-sulfonic) acid (ABTS) from Alfa Aesar (Kandel, Germany). 

Solvents used were ethanol (EtOH) (>99.8%, Honeywell Riedel-de Haën^TM^, Germany), acetonitrile (ACN, HPLC grade, Fisher Scientific, UK), formic acid (FA) (98–100% *w/w*, Merck, Darmstadt, Germany) and hydrochloric acid (32% *w/w*, Merck, Darmstadt, Germany). Ultrapure water was obtained from a Milli-Q system (Merck Millipore). Nylon syringe filters (13 mm, 0.22 μm) were from Filter-Lab^®^ (Filtros Anoia, Sant Pere de Riudebitlles, Barcelona, Spain). 

### 2.2. Samples

Olive oil and winery residue samples were provided by Spanish industries. Olive oil residue sampling was performed in the period between November 2018 and February 2019. Samples O1, O2, O3, and O4 were all olive pomace paste (named alperujo) from different olive oil producers. Wine residues were obtained throughout 2017 and 2018 from local wineries. The characteristics of the samples are summarized in Table 1. Both olive oil and winery residues were stored in the freezer at −20 °C.

### 2.3. Instrumentation and Equipment

HPLC–UV system: Agilent Series 1200 system (Agilent Technologies, Palo Alto, CA, USA), equipped with a quaternary pump, an automatic injection system, a diode array detector (DAD), and Agilent ChemStation software for data analysis. Ultra-high performance liquid chromatography high resolution mass spectrometry (UHPLC–HRMS) system: Accela (Thermo Scientific, Hemel Hempstead, UK) equipped with a quaternary pump, a thermostatic autosampler, a DAD, and coupled to an LTQ Orbitrap Velos mass spectrometer (Thermo Scientific, Hemel Hempstead, UK) with an ESI source and Xcalibur Qual Browser software for HRMS data handling. Spectrophotometer: Double beam Perkin Elmer UV/Vis/NIR Lambda 19 (Waltham, MA, USA) with QS quartz glass high performance cuvettes (10 mm optical path) from Hellma Analytics (Jena, Germany). UAE system: Ultrasonic bath (Branson 5510, Danbury CT, USA), with a frequency of 42 kHz and power of 135 W. MAE system: Milestone Microwave Labstation (Ethos E, Milestone, Shelton, CT, USA). PLE system: Accelerated solvent extractor Dionex ASE 350 (Dionex Corp., Sunnyvale, CA, USA) equipped with 5 mL stainless steel extraction cells. Other: Labofuge 400 centrifuge (Heraeus, Hanau, Germany), Vibra mix R agitator (OVAN, Badalona, Spain).

### 2.4. Procedures

#### 2.4.1. High Performance Liquid Chromatography Analysis with Ultraviolet Detection (HPLC–UV)

A Kinetex C_18_ column (Phenomenex, 100 mm × 4.6 mm × 2.6 μm, Torrance, CA, USA) was used for chromatographic analysis. Ultrapure water with 0.1% FA (A), and ACN (B) were used as mobile phase components. The gradient program for olive oil residues analysis was as follows: 0 min, 5% B; 0–38 min, 35% B; 38–40 min, 90% B; 40–42 min, 90% B; 42–42.2 min, 5% B; 42.2–50 min, 5% B; for wine residues the program was 0 min, 5% B; 0–38 min, 45% B; 38–40 min, 90% B; 40–42 min, 90% B; 42–42.2 min, 5% B; 42.2–50 min, 5% B. The flow rate was 0.4 mL min^−1^ and the injection volume 5 μL. Chromatograms were recorded at 280, 310, 370, and 550 nm. The total polyphenol content (TPC) was estimated from the total peak area in the chromatograms at 280 nm, in the time range between 5 and 36 min. TPC was expressed in terms of mg of gallic acid equivalent (GAE) per g of fresh weight (mg GAE g^–1^).

#### 2.4.2. Ultra-High Performance Liquid Chromatography High Resolution Mass Spectrometry (UHPLC–HRMS)

The chromatographic separation was carried out with a Kinetex C_18_ column (Phenomenex, 100 mm × 4.6 mm × 2.6 μm, Torrance, CA, USA). Ultrapure water with 0.1% FA (A) and ACN (B) were used as mobile phases. The gradient program was 0 min, 5% B; 0–25 min, 50% B; 25–27 min, 90% B; 27–29 min, 90% B; 29–29.2 min, 5% B; 29.2–39 min, 5% B; at a flow rate of 0.8 mL min^−1^ and an injection volume of 5 μL. For MS detection, the electrospray ionization (ESI) in negative mode was used. MS spectra were acquired in the *m/z* range 100 to 1500 at a mass resolution of 30,000 full width at half-maximum (FWHM) at *m/z* 200. Operation parameters were as follows: source voltage, 4 kV; sheath gas, 20 (arbitrary units); auxiliary gas, 10 (arbitrary units); sweep gas, 2 (arbitrary units); and capillary temperature, 275 °C. Automatic gain control (AGC) target 5 × 10^5^ for MS mode was applied.

#### 2.4.3. Determination of Antioxidant Indexes

Folin–Ciocalteu (FC) and ABTS methods have been described in detail elsewhere [42]. Briefly, spectrophotometric measurements were carried out at the selected wavelengths (765 nm for FC and 734 nm for ABTS) using a double-beam system in which test and blank solutions were placed in the sample and reference holders, respectively. Following the typical ways of expressing these antioxidant indexes, FC results were given as mg GAE g^−1^ fresh weight and ABTS results as mg Trolox g^−1^ fresh weight. 

#### 2.4.4. Ultrasound Assisted Extraction (UAE)

According to our previous experience in the laboratory with a wide range of samples, extraction time was set at 30 min and the sample (g):solvent (mL) ratio at 1:20. Solvent composition was varied according to a 3^2^ factorial design, in which 2 factors (%EtOH and %HCl) were investigated at three levels (40, 60, and 80% EtOH; 0, 0.1, and 0.5% HCl). A total of 9 experiments were performed in triplicate (Table S1, Supplementary Material). 

Thus, 1 g of sample and 20 mL of extraction solvent were placed into a 45 mL Falcon tube and vortexed. The tubes were placed into an ultrasonic bath at room temperature (20 °C) for 30 min, the final temperature being 24 °C. Then, the samples were centrifuged for 15 min at 3500 rpm, filtered with nylon syringe filters, and stored at 4 °C until analysis by HPLC–UV.

#### 2.4.5. Microwave Assisted Extraction (MAE)

MAE, using water–ethanol mixtures, was investigated, and factorial designs were applied to evaluate the influence of EtOH percentage and temperature (at three levels) and time (at two levels). Levels for each factor were as follows: %EtOH (20, 50, and 80% EtOH), temperature (60, 90, and 120 °C), and extraction time (5 and 15 min), thus involving a total of 18 experiments. One gram of sample was placed in an MAE vessel, and 20 mL of extraction solvent was added. Stirring was set at 35% with a magnet inside each vessel. After extraction, samples were cooled down to room temperature and transferred to a Falcon tube. Finally, the samples were centrifuged and filtered with nylon syringe filters, and the extracts were stored at 4 °C until analysis by HPLC–UV. Experiments were performed in triplicate. 

#### 2.4.6. Pressurized Liquid Extraction (PLE)

PLE experiments were performed using the water–ethanol system as the extracting solvent. The effect of solvent composition (40, 60, and 80% EtOH), temperature (80, 100, and 120 °C), extraction time (5, 10, and 15 min), and number of cycles (1 and 2) was investigated. One gram of sample was mixed with 2 g of diatomaceous earth (Thermo Scientific) and placed in a 5 mL stainless steel extraction cell containing a fiberglass filter (Thermo Scientific) at the bottom. Prior to starting a PLE series, the system was rinsed with the extraction solvent in order to prevent contamination. Preheating time of cells was 5 min, extraction pressure was constant at 1500 psi, and the flush volume was set at 60%. To prevent polyphenolic oxidation during extraction, the cells were purged with nitrogen for 60 s. The extracted samples were collected into 20 mL glass vials and then transferred to 15 mL Falcon tubes. The samples were centrifuged, filtered, and stored at 4 °C until analysis by HPLC–UV. Each extraction experiment was performed in triplicate.

### 2.5. Data Analysis 

Response surface methodology (RSM) was used to evaluate the influence of the experimental variables on polyphenol extraction. Response surface plots were obtained with MATLAB^©^ R2012a (The MathWorks, Inc., Natick, MA, USA). Two-factor analysis of variance (ANOVA) with replication at 95% confidence level (*p* < 0.05) was also applied to test statistically the significance of effects. ANOVA was performed with Microsoft Excel 2019.

## 3. Results and Discussion

### 3.1. Ultrasound Assisted Extraction (UAE)

The performance of the ethanol–water–hydrochloric acid system in the extraction of polyphenols from olive pomace (sample O1) and lees (sample W4) samples was evaluated by UAE. Both water and ethanol are well-suited solvents in terms of applications involving food, cosmetic, and nutraceutical industries, and water–ethanol mixtures are widely proposed in the literature for the extraction of polyphenols from different matrices. It has been also reported that, in general, the acidity of the medium has a positive effect on the polyphenol extraction yield [41,43,44], so hydrochloric acid was included as a variable to be assessed.

In this study, ethanol percentage was varied 40–80%, while the concentration of hydrochloric acid was varied 0–0.5%, and experiments were performed according to the procedure described in Section 2.4.4.

**Figure 1:**
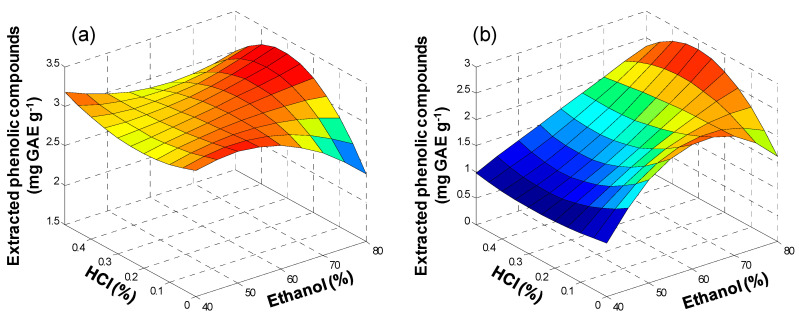
Response surface plots for extracted phenolic compounds (mg GAE g^−1^) in (**a**) olive pomace and (**b**) lees filters by UAE as a function of ethanol (%) and HCl (%) concentration.

Results are given in Table S1 (Supplementary Material), and response surfaces for TPC extraction, expressed in terms of mg GAE g^−1^ sample, are shown in Figure 1. In the case of olive pomace (Figure 1a), a quite flat surface was obtained, indicating that the composition of the solvent had a minor influence on the extraction within the explored range. ANOVA study of the results (Table S5, Supplementary Material) confirmed that, under the conditions tested, there was no significant effect of the concentration of either ethanol (*p* = 0.06) or HCl (*p* = 0.90) on the extraction recovery. 

In the literature, the extraction of polyphenols from olive pomace by UAE has been carried out using diverse percentages of ethanol, up to 90% [25]. Results presented here show that, in the explored ranges, the percentage of ethanol was not a critical issue, and hydrochloric acid was not necessary to enhance extraction.

Conversely, in the case of lees filters (Figure 1b), there was a clear influence of ethanol concentration on TPC recovery. Thus, extraction improved significantly (*p* = 1.2 × 10^−10^) when the concentration of ethanol was above 40%. This is in accordance with the 50% EtOH proposed by other authors for polyphenol extraction from grape pomace [39] and grape skin [40]. On the other hand, there was no influence of the HCl concentration (*p* = 0.64), which agrees with results reported by Bachtler and Bart [41], about the effect of HCl concentration on polyphenol extraction from vine leaves.

Taking into account that HCl did not show a significant effect on polyphenol extraction by UAE, neither from olive pomace nor lees filters, subsequent studies on MAE and PLE focused on the water–ethanol system, without considering the addition of HCl.

**Figure 2:**
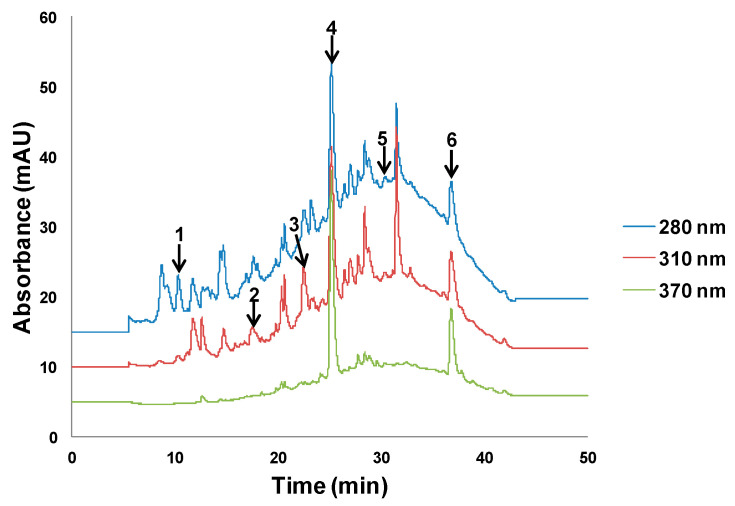
Olive pomace chromatograms at 280, 310, and 370 nm. Peak assignment: 1—3-hydroxytyrosol, 2—caffeic acid, 3—*p*-coumaric acid, 4—rutin, 5—oleuropein, 6—luteolin.

**Figure 3:**
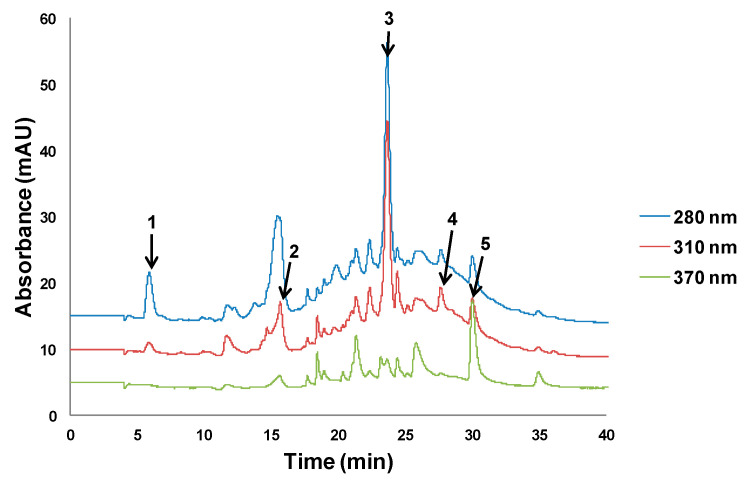
Lees filter chromatograms at 280, 310, and 370 nm. Peak assignment: 1—gallic acid, 2—caffeic acid, 3—hesperidin, 4—resveratrol, 5—quercetin.

The different behavior of the two matrices was attributed to the differences in polyphenol composition, which is connected to the chromatographic profiles of both kinds of samples. Chromatograms of the olive pomace extracts (Figure 2) were more complex than those of lees extracts (Figure 3). In olive pomace, extracts were rich in compounds with a wide range of polarities, so that increasing the ethanol percentage in the extraction solvent may have contributed to improvement in the recovery of less polar compounds while decreasing the recovery of polar ones. The presence of oleuropein and luteolin, characteristic compounds in olives, was confirmed by UHPLC–HRMS, as well as 3-hydroxytyrosol, caffeic acid, *p*-coumaric acid, and rutin. In contrast, lees extracts were abundant in medium polarity compounds, which were better extracted when the percentage of organic solvent was increased. The presence of gallic acid, caffeic acid, hesperidin, resveratrol, and quercetin was confirmed by UHPLC–HRMS.

### 3.2. Microwave Assisted Extraction (MAE)

Polyphenols extraction by MAE from olive pomace and lees filters was investigated using water–ethanol mixtures. Conditions reported in the literature for the extraction of polyphenols by MAE are diverse. For instance, Habibi et al. [26] proposed a mixture of EtOH:water 60:40 (*v/v*) as extraction solvent, 220 W of microwave power, and 12 min of extraction time, whereas Jurmanović et al. [27], established as optimum MAE conditions EtOH:water 20:80 (*v/v*), 700 W of microwave power, and 10 min of extraction time. 

In MAE, apart from issues dealing with the affinity among solvent and analytes, the polarity of the solvent plays an important role, since the absorption of microwave radiation is more efficient in polar media, and thus the extraction recovery might be improved. In this study, ethanol was varied between 20 and 80%, a wider range than in UAE. For temperature, the range was 60–120 °C, and for extraction time, 5 and 15 min were considered. Experiments were performed following the procedure described in Section 2.4.5.

**Figure 4:**
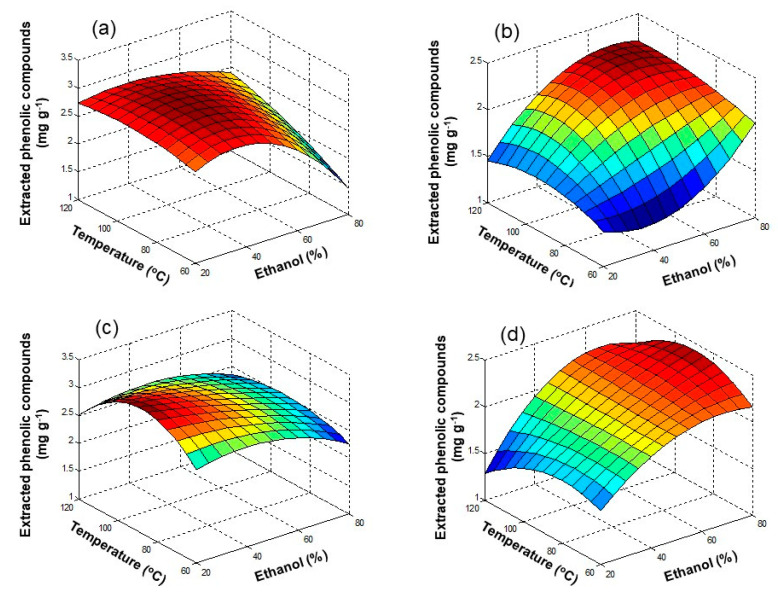
Response surface plots for extracted phenolic compounds (mg GAE g^−1^) in olive pomace and lees filters by MAE as a function of ethanol concentration (%), temperature (°C), and extraction time (min): (**a**) olive pomace, 5 min; (**b**) lees filters, 5 min; (**c**) olive pomace, 15 min; (**d**) lees filters, 15 min.

Results are shown in Figure 4 and in Table S2 (Supplementary Material). In the case of olive pomace (Figure 4a,c), TPC improved by increasing the ethanol concentration from 20 to 50%, and temperature from 60 to 90 °C. However, when increasing ethanol concentration from 50 to 80%, some decrease in TPC values was observed; at 120 °C TPC decayed, which was attributed to degradation of polyphenols in the samples at higher temperatures. ANOVA results confirmed that there was a significant effect of ethanol concentration (*p* = 1.1 × 10^−9^) and temperature (*p* = 8.5 × 10^−5^) on the extraction of polyphenols. Conversely, regarding extraction time, no significant improvement was observed when increasing time from 5 to 15 min (*p* = 0.21). 

In the case of lees filters (Figure 4b,d), a similar trend with respect to olive pomace samples was observed. The TPC improved with the increase of ethanol concentration, from 20 to 50%, and with temperature, from 60 to 90 °C. Nevertheless, by increasing the ethanol concentration from 50 to 80%, TPC remained almost the same, and at 120 °C, TPC decreased because of thermal degradation. Concerning extraction time, no significant effect was observed when increasing time from 5 to 15 min. ANOVA (Table S5, Supplementary Material) confirmed that both ethanol concentration and temperature had a significant influence on the polyphenol extraction (*p* = 2.4 × 10^−8^ and *p* = 1.0 × 10^−4^, respectively), but not extraction time (*p* = 0.18). Garrido et al. [32] proposed the use of 48% ethanol in water and 10 min of extraction time at 25 °C for MAE extraction of phenolic compounds from Chardonnay grape marc; they also reported degradation at high temperatures, which is consistent with our results.

### 3.3. Pressurized Liquid Extraction (PLE)

Finally, polyphenol extraction by PLE from olive pomace and lees filters samples using water–ethanol mixtures was explored. 

EtOH percentage and temperature were varied at three levels (ethanol: 40, 60, and 80%; temperature: 80, 100, and 120 °C), with an extraction static time of 5 min and 1 cycle, with a total of 9 experiments for each matrix, which were performed in triplicate, following the procedure described in Section 2.4.6. 

**Figure 5:**
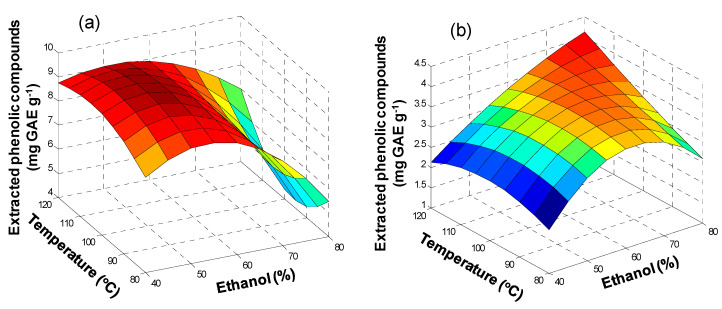
Response surface plots for extracted phenolic compounds (mg GAE g^−1^) in (**a**) olive pomace and (**b**) lees filters by PLE as a function of ethanol concentration (%) and temperature (°C).

Results are shown in Figure 5 and in Table S3 (Supplementary Material). In the case of olive pomace (Figure 5a), results showed that from 40 to 60% ethanol, there was some improvement of the extraction, but at 80% EtOH, there was a decrease in TPC. Regarding extraction temperature, no relevant influence was observed. ANOVA results confirmed that under the conditions tested, the effect of the ethanol concentration on TPC extraction was significant (*p* = 9.2 × 10^−9^), while the influence of temperature was irrelevant (*p* = 0.12).

These results agree with extraction conditions reported by other authors for PLE extraction of polyphenols from olive pomace [19] or olive leaves [20], which proposed ethanol:water 50:50 (*v/v*) as extraction solvent, and different temperature conditions (120 °C for pomace and 80 °C for leaves).

A range of conditions for the PLE extraction time or the number of cycles can be found in the literature. For instance, Putnik et al. [20] proposed 2 cycles of 5 min of extraction time (ethanol:water 50:50 (*v/v*); 80 °C) for polyphenol extraction from olive leaves, whereas Xynos et al. [14] applied 3 extraction cycles of 5 min (EtOH at 190 °C). In this study, PLE experiments with 1 and 2 cycles, and 5, 10, and 15 min extraction times were performed (ethanol:water 50:50 (*v/v*), T = 100 °C). 

Results are shown in Table S4 (Supplementary Material). For olive pomace, it was concluded that one extraction cycle was enough, since no advantages were observed when adding an extra cycle (*p* = 0.57). Regarding extraction time, there was an effect on TPC (*p* = 0.02); the highest TPC values were obtained at 5 min. The decrease of TPC at longer extraction time was probably due to a degradation of polyphenols. In this sense, Putnik et al. [20] also observed a TPC decrease with increasing extraction time. 

In the case of lees filters (Figure 5b), TPC increased with ethanol concentration from 40 to 60% but decreased at 80%. Regarding extraction temperature, TPC increased from 80 to 100 °C and decreased at 120 °C, except for 80% EtOH. ANOVA tests confirmed that ethanol concentration and temperature had significant influence in the polyphenol extraction (*p* = 1.7 × 10^−7^ and *p* = 7.4 × 10^−3^, respectively). No significant effects either of the number of cycles or extraction time (*p* = 0.07 and *p* = 0.39, respectively) were found.

### 3.4. Extraction of Polyphenols from Olive Oil Mill and Winery Wastes

Taking into account the results of the previous extraction experiments, the ethanol:water 50:50 (*v/v*) mixture was selected as the extraction solvent for both kinds of residues and the three techniques. Table 2 summarizes the proposed experimental conditions for TPC extraction. Finally, it was decided to apply the three techniques, under the selected conditions, to a set of diverse residues from olive oil and wine companies. 

**Figure 6:**
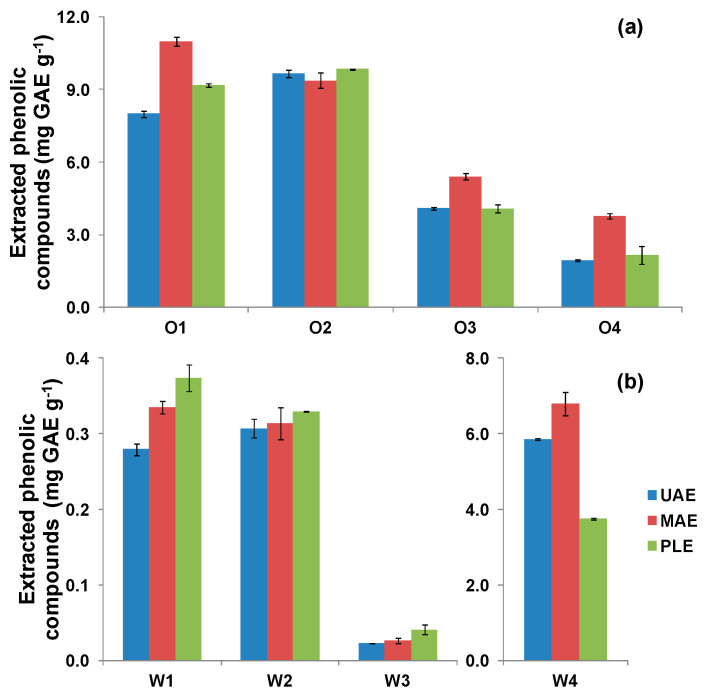
Extraction techniques comparison in olive mill (**a**) and winery (**b**) residues.

Figure 6a shows the results obtained for samples related to the olive oil sector. In a global sense, there were not large differences between the results of the extraction techniques, although it can be observed that MAE was the most efficient. ANOVA analysis of the results (Table S6, Supplementary Material) confirmed the significant differences between the three extraction techniques (*p* = 9.3 × 10^−8^) under the studied conditions and also pointed out that there was interaction between samples and techniques (*p* = 2.3 × 10^−4^), i.e., the performance of the technique depended on the sample. With regard to winery wastes, again there were not large differences between the performance of the extraction techniques (Figure 6b), but PLE provided the highest TPC values, except for the W4 sample, for which MAE was the most efficient. ANOVA (Table S7, Supplementary Material) confirmed the significant differences between the extraction techniques (*p* = 5.7 × 10^−7^) and that there was sample–technique interaction (*p* = 6.2 × 10^−10^). Compared with similar cases using ethanol:water 50:50 (*v/v*), Drosou et al. [39] and Caldas et al. [40] found that UAE was more efficient than MAE for the extraction of polyphenols from grape pomace and grape skin samples, respectively. 

Overall, in this study MAE provided higher efficiency for olive oil wastes and PLE for winery wastes, but results from UAE were also satisfactory. In this context, Talmaciu et al. [45], in a comparative investment costs study considering different extraction techniques, such as MAE and UAE and supercritical fluid extraction, concluded that UAE is the one that requires lower capital and operational costs. Instead, in a study about polyphenols extraction from red wine pomace, Vega et al. [46], carried out a techno-economic and life cycle assessment, and concluded that PLE not only had higher capital expenses, but also higher operational expenses and environmental concerns. Hence, considering extraction performance and simplicity, but also investment and operational costs, we propose UAE for further scaling up.

Focusing on the phenolic yield of the different samples, it can be noticed that the phenolic content in olive pomace samples was, in general, higher than that of winery residues. Additionally, there were significant differences between the yields of olive pomaces (*p* = 1.1 × 10^−22^) and also between the yields of winery residues (*p* = 1.7 × 10^−23^). Concerning olive pomace, since all the samples were processed by using similar treatment stages, the differences in yields were mainly attributed to geographical and varietal issues. Olive oils from southern Spanish regions are richer in phenolic compounds due to the higher levels of hydric stress induced by the more severe climatic conditions [47]. In addition, oils from varieties such as picual are noticeably richer in polyphenols than arbequina counterparts, with concentrations ca. two-fold higher [48]. The behavior found in olive oils can be reasonably extrapolated to their corresponding residues. This agrees with the results found here, i.e., samples from northern Spanish areas (O3, O4) and/or those produced with arbequina olives contain lower phenolic concentrations. Regarding winery residues, the yield for sample W3 (lees filters, white wine, chardonnay, sauvignon blanc, and xarel·lo varieties) was clearly lower than those of the rest of the winery samples, which is related to the lower phenolic contents of lees from white wines compared to lees of reed wines [49]. Conversely, the phenolic yield of sample W4 (lees filters, red wine, garnacha, tempranillo, cabernet sauvignon, and cariñena) was the highest, in the range of winery residue samples, and thus the filters of lees of red wine production appeared to be a good source for the recovery of polyphenols.

Finally, we evaluated the antioxidant activity of extracts from the samples using Folin–Ciocalteu and ABTS assays [50,51]. The results, as well as those from HPLC, are collected in Table 3.

There was no clear correlation between the results of the different methods. The main reason is that the three methods were based on different approaches. The results of HPLC–UV were obtained from the absorbance measured at 280 nm, which in these types of samples was mainly due to polyphenols. The Folin–Ciocalteu method is based on a redox reaction, while the ABTS method is based on a radical reaction, and in both assays, different polyphenols show different sensitivity [42]. In any case, all the assays indicated that extracts of residue samples from olive oil production had higher antioxidant activity levels than the extracts of the winery residue samples, with the exception of sample W4. 

## 4. Conclusions

The extraction of polyphenols by UAE, MAE, and PLE from a set of representative residues of the production of olive oil and wine was explored using ethanol:water mixtures. It was concluded that, within the experimental domain of the study, the extraction performance was not dependent on the hydrochloric acid concentration. For all the techniques and both matrices, the ethanol percentage influenced TPC values, except for the UAE–olive pomace system, where no relevant effect was observed in the explored range. Ethanol:water 50:50 (*v/v*) was identified as a suitable mixture for the extraction of polyphenols, being compatible with food, nutraceutical, and cosmetic applications. Temperature can enhance extraction yields in MAE and PLE, but values above 100 °C should be avoided to prevent thermal degradation. Overall, MAE showed higher extraction efficiency for olive pomace samples, while PLE was more efficient for winery residues. Nevertheless, UAE also provided good performance with both types of samples, and because of its simplicity, as well as the lower capital and operational costs reported [45,46], it is here proposed for further scaling up evaluation.

Olive pomace extracts were rich in compounds with a wide range of polarities, while lees extracts were abundant in medium polarity compounds. UHPLC–HRMS experiments confirmed the presence of 3-hydroxytyrosol, caffeic acid, *p*-coumaric acid, rutin, oleuropein, and luteolin in olive pomace extracts, whereas the presence of gallic acid, caffeic acid, hesperidin, resveratrol, and quercetin was verified in lees extracts.

The selection of suitable sources for developing polyphenol recovery processes depends, among other issues, on the polyphenol yield. In general terms, olive pomaces have a high content of polyphenols, which depend on the olive variety and the climatic conditions; concerning the availability of olive pomace, it is limited to four months per year. Regarding winery wastes, they provide lower yields, although lees filters of red wine showed a similar yield to that obtained with olive pomaces. Lees filters are available the entire year, but their production is rather low compared to olive pomace or wine lees. As a final point to be considered, the Spanish regulation on winery waste management promotes the valorization for the production of ethanol in which winery wastes are macerated with water and then distillated to recover ethanol. Thus, valorization routes based on polyphenols may be linked to their recovery before the distillation stage.

## Figures and Tables

**Table 1:** Olive oil and winery residue samples.

Sample Code	Origin	Sample Type	Characteristics	Variety
**Olive oil residues**				
O1	Córdoba (Spain)	Olive pomace	Solid (paste)	Hojiblanca and Picual
O2	Córdoba (Spain)	Olive pomace	Solid (paste)	Hojiblanca, Picual, and Arbequina
O3	Huesca (Spain)	Olive pomace	Solid (paste)	Verdeña
O4	Lleida (Spain)	Olive pomace	Solid (paste)	Arbequina
**Wine residues**				
W1	Ciudad Real (Spain)	Wine lees	Solid (paste)	Red wine (Tempranillo)
W2	Ciudad Real (Spain)	Wine lees	Solid (paste)	Red wine (Tempranillo)
W3	Barcelona (Spain)	Diatomaceous earth filter media (lees filters)	Solid (paste)	White wine (Chardonnay, Sauvignon Blanc, Xarel·lo)
W4	Barcelona (Spain)	Diatomaceous earth filter media (lees filters)	Solid (paste)	Red wine (Garnacha, Tempranillo, Cabernet Sauvignon, Cariñena)

**Table 2:** Selected conditions, for polyphenol extraction from olive oil and wine industries wastes.

Technique	Solvent (*v*/*v*)	Temperature (°C)	Time (min)	Cycles
UAE	EtOH:water 50:50	Room temperature (20)	30	-
MAE	EtOH:water 50:50	90	5	-
PLE	EtOH:water 50:50	100	5	1

**Table 3:** Analysis of UAE extracts from olive oil and wine production wastes by HPLC–UV Folin–Ciocalteu, and ABTS assays.

Sample Code	HPLC–UV	Folin–Ciocalteu	ABTS
	(mg GAE/g)		(mg Trolox/g)
O1	8.00 ± 0.12	2.77 ± 0.02	6.62 ± 0.23
O2	9.67 ± 0.14	8.05 ± 0.59	31.63 ± 0.46
O3	4.09 ± 0.06	4.22 ± 0.32	8.15 ± 0.34
O4	1.93 ± 0.03	2.05 ± 0.17	2.41 ± 0.11
W1	0.28 ± 0.01	1.98 ± 0.25	2.22 ± 0.03
W2	0.31 ± 0.01	0.88 ± 0.06	0.90 ± 0.10
W3	0.02 ± 0.01	0.08 ± 0.01	0.15 ± 0.01
W4	5.85 ± 0.03	1.50 ± 0.16	7.18 ± 0.10

## Supplementary Materials

The following are available online at https://www.mdpi.com/2076-3921/9/11/1074/s1, Table S1: TPC in olive pomace and lees filters recovered by UAE (average ± standard deviation). Table S2: TPC in olive pomace and lees filters recovered by MAE (average ± standard deviation). Table S3: TPC in olive pomace and lees filters recovered by PLE: solvent and temperature (average ± standard deviation). Table S4: TPC in olive pomace and lees filters recovered by PLE: cycles and time (average ± standard deviation). Table S5: Effect of experimental variables on the extraction of polyphenols: summary of p-values from ANOVA. Table S6: ANOVA table for olive oil residues and extraction techniques. Table S7: ANOVA table for winery residues and extraction techniques.
