# Supplementary material for: Olive Mill and Winery Wastes as Viable Sources of Bioactive Compounds: A Study on Polyphenols Recovery
Source: Antioxidants (Basel). 2020 Nov 1;9(11):1074. doi: 10.3390/antiox9111074 (PMC7694004; doi:10.3390/antiox9111074)
Supplement: Supplementary file 1 [file antioxidants-09-01074-s001.zip › antioxidants-948960-supplementary.docx]

Supplementary material

**Table S1.** TPC in olive pomace and lees filters recovered by UAE (average ± standard deviation).

| **TPC (mg GAE g^-1^)** | | | |
| --- | --- | --- | --- |
| **EtOH (%)** | **HCl (%)** | **Olive pomace** | **Lees filters** |
| 40 | 0 | 3.10 ± 0.12 | 1.02 ± 0.04 |
| 40 | 0.1 | 3.05 ± 0.03 | 0.93 ± 0.01 |
| 40 | 0.5 | 3.14 ± 0.17 | 0.99 ± 0.03 |
| 60 | 0 | 3.06 ± 0.06 | 2.37 ± 0.04 |
| 60 | 0.1 | 3.10 ± 0.05 | 2.01 ± 0.27 |
| 60 | 0.5 | 2.78 ± 0.06 | 1.68 ± 0.34 |
| 80 | 0 | 2.79 ± 0.01 | 1.61 ± 0.04 |
| 80 | 0.1 | 2.87 ± 0.04 | 2.27 ± 0.06 |
| 80 | 0.5 | 2.99 ± 0.01 | 2.34 ± 0.07 |

**Table S2.** TPC in olive pomace and lees filters recovered by MAE (average ± standard deviation).

| **EtOH (%)** | **Temperature (°C)** | **Extraction time** | | | |
| --- | --- | --- | --- | --- | --- |
|  |  | **Olive pomace TPC**  **(mg GAE g^-1^)** | | **Lees filters TPC**  **(mg GAE g^-1^)** | |
|  |  | **5 min** | **15 min** | **5 min** | **15 min** |
| 20 | 60 | 2.64 ± 0.07 | 2.67 ± 0.02 | 1.37 ± 0.07 | 1.57 ± 0.03 |
|  | 90 | 2.80 ± 0.01 | 3.22 ± 0.22 | 1.55 ± 0.08 | 1.64 ± 0.05 |
|  | 120 | 2.73 ± 0.08 | 2.50 ± 0.05 | 1.45 ± 0.01 | 1.28 ± 0.04 |
| 50 | 60 | 2.71 ± 0.05 | 2.75 ± 0.01 | 1.35 ± 0.05 | 2.10 ± 0.16 |
|  | 90 | 2.91 ± 0.01 | 2.92 ± 0.06 | 2.10 ± 0.02 | 2.11 ± 0.03 |
|  | 120 | 2.77 ± 0.07 | 2.67 ± 0.09 | 2.14 ± 0.01 | 2.12 ± 0.08 |
| 80 | 60 | 1.46 ± 0.07 | 2.26 ± 0.05 | 2.01 ± 0.01 | 2.15 ± 0.06 |
|  | 90 | 2.04 ± 0.09 | 2.51 ± 0.02 | 2.17 ± 0.01 | 2.42 ± 0.16 |
|  | 120 | 2.39 ± 0.06 | 2.35 ± 0.05 | 2.21 ± 0.07 | 2.14 ± 0.08 |

**Table S3**. TPC in olive pomace and lees filters recovered by PLE: solvent and temperature (average ± standard deviation).

| **EtOH (%)** | **Temperature (°C)** | **TPC (mg GAE g^-1^)** | |
| --- | --- | --- | --- |
|  |  | **Olive pomace** | **Lees filters** |
| 40 | 80 | 7.96 ± 0.28 | 2.05 ± 0.09 |
| 40 | 100 | 9.02 ± 0.09 | 2.47 ± 0.16 |
| 40 | 120 | 8.73 ± 0.23 | 2.13 ± 0.08 |
| 60 | 80 | 8.63 ± 0.28 | 3.45 ± 0.06 |
| 60 | 100 | 8.92 ± 0.24 | 3.64 ± 0.06 |
| 60 | 120 | 8.89 ± 0.02 | 3.33 ± 0.08 |
| 80 | 80 | 5.45 ± 0.38 | 2.58 ± 0.28 |
| 80 | 100 | 4.42 ± 0.31 | 3.31 ± 0.33 |
| 80 | 120 | 7.01 ± 0.30 | 4.12 ± 0.02 |

**Table S4.** TPC in olive pomace and lees filters recovered by PLE: cycles and time (average ± standard deviation).

| **Cycles** | **Time (min)** | **TPC (mg GAE g^-1^)** |  |
| --- | --- | --- | --- |
|  |  | **Olive pomace** | **Lees filters** |
| 1 | 5 | 9.52 ± 0.20 | 3.58 ± 0.02 |
| 1 | 10 | 7.46 ± 0.26 | 3.32 ± 0.15 |
| 1 | 15 | 7.80 ± 0.05 | 3.62 ± 0.02 |
| 2 | 5 | 8.98 ± 0.21 | 3.88 ± 0.25 |
| 2 | 10 | 8.04 ± 0.11 | 4.12 ± 0.01 |
| 2 | 15 | 6.88 ± 0.22 | 5.34 ± 0.98 |
| 3 | 5 | - | 3.62 ± 0.36 |
| 3 | 10 | - | 4.21 ± 0.13 |
| 3 | 15 | - | 4.04 ± 0.04 |

**Table S5.** Effect of experimental variables on the extraction of polyphenols: summary of p-values from ANOVA.

| **Technique** | **% EtOH** | | **% HCl** | | **Temperature** | | **Time** | | **Number of cycles** | |
| --- | --- | --- | --- | --- | --- | --- | --- | --- | --- | --- |
|  | **Olive pomace** | **Lees filters** | **Olive pomace** | **Lees filters** | **Olive pomace** | **Lees filters** | **Olive pomace** | **Lees filters** | **Olive pomace** | **Lees filters** |
| UAE | 0.0608 | 1.16E-10 | 0.8978 | 0.6444 | - | - | - | - | - | - |
| MAE | 1.13E-09 | 2.40E-08 | - | - | 8.47E-05 | 0.0001 | 0.2058 | 0.1769 | - | - |
| PLE | 9.20E-09 | 1.73E-07 | 0.0627 | 0.2790 | 0.1220 | 0.0074 | 0.0226 | 0.3915 | 0.5737 | 0.0682 |

**Table S6.** ANOVA table for olive oil residues and extraction techniques.

| **Variations origin** | **Sum of squares** | **Degrees of freedom** | **Mean square** | **F value** | **Probability** | **Critical value for F** |
| --- | --- | --- | --- | --- | --- | --- |
| Sample | 352.39 | 3 | 117.46 | 597.80 | 1.13E-22 | 3.01 |
| Extraction technique | 13.47 | 2 | 6.73 | 34.27 | 9.26E-08 | 3.40 |
| Interaction | 8.15 | 6 | 1.36 | 6.91 | 2.35E-04 | 2.51 |
| Within the group | 4.72 | 24 | 0.20 |  |  |  |
| Total | 378.72 | 35 |  |  |  |  |

**Table S7.** ANOVA table for winery residues and extraction techniques.

| **Variations origin** | **Sum of squares** | **Degrees of freedom** | **Mean square** | **F value** | **Probability** | **Critical value for F** |
| --- | --- | --- | --- | --- | --- | --- |
| Sample | 186.71 | 3 | 62.24 | 700.99 | 1.71E-23 | 3.01 |
| Extraction technique | 4.93 | 2 | 2.47 | 27.78 | 5.67E-07 | 3.40 |
| Interaction | 15.68 | 6 | 2.61 | 29.44 | 6.17E-10 | 2.51 |
| Within the group | 2.13 | 24 | 0.09 |  |  |  |
| Total | 209.46 | 35 |  |  |  |  |
